# Flavonoid Accumulation Varies in *Medicago truncatula* in Response to Mercury Stress

**DOI:** 10.3389/fpls.2022.933209

**Published:** 2022-07-07

**Authors:** Gerardo Alvarez-Rivera, Aurora Sanz, Alejandro Cifuentes, Elena Ibánez, Timothy Paape, M. Mercedes Lucas, José J. Pueyo

**Affiliations:** ^1^Laboratory of Foodomics, CIAL-CSIC, Institute of Food Science Research, Madrid, Spain; ^2^Institute of Agricultural Sciences, ICA-CSIC, Madrid, Spain; ^3^Brookhaven National Laboratory, Upton, NY, United States

**Keywords:** *Medicago truncatula*, flavonoids, mercury, metabolomics, heavy metal stress, LC-QTOF

## Abstract

Mercury (Hg) contamination is increasing worldwide in both wild ecosystems and agricultural soils due to natural processes, but mostly to anthropic activities. The molecular mechanisms involved in Hg toxicity and tolerance in plants have been extensively studied; however, the role of flavonoids in response to Hg stress remains to be investigated. We conducted a metabolomic study to analyze the changes induced at the secondary metabolite level in three Hg-tolerant and one Hg-sensitive *Medicago truncatula* cultivars. A total of 46 flavonoid compounds, classified into five different flavonoid families: anthocyanidins, flavones, isoflavones, pterocarpan flavonoids, and flavanones, along with their respective glycoconjugate derivatives, were identified in leaf and root tissues. The synthesis of free isoflavones, followed by monoglycosylation and further malonylation was shown to be characteristic of root samples, whereas higher glycosylation, followed by further acylation with coumaric and ferulic acid was characteristic of leaf tissues. While minor changes were observed in leaves, significant quantitative changes could be observed in roots upon Hg treatment. Some flavonoids were strongly upregulated in roots, including malonylglucosides of biochanin A, formononetin and medicarpin, and aglycones biochanin, daidzein, and irisolidone. Hg tolerance appeared to be mainly associated to the accumulation of formononetin MalGlc, tricin GlcAGlcA, and afrormosin Glc II in leaves, whereas aglycone accumulation was associated with tolerance to Hg stress in roots. The results evidence the alteration of the flavonoid metabolic profile and their glycosylation processes in response to Hg stress. However, notable differences existed between varieties, both in the basal metabolic profile and in the response to treatment with Hg. Overall, we observed an increase in flavonoid production in response to Hg stress, and Hg tolerance appeared to be associated to a characteristic glycosylation pattern in roots, associated with the accumulation of aglycones and monoglycosylated flavonoids. The findings are discussed in the context of the flavonoid biosynthetic pathway to provide a better understanding of the role of these secondary metabolites in the response and tolerance to Hg stress in *M. truncatula*.

## Introduction

Agricultural soils are increasingly affected by contamination by heavy metals as a consequence of sewage irrigation practices, atmospheric deposition, use of livestock manures, soil amendments, and various agrophytochemicals (Peng et al., 2019). Heavy metals may also be present naturally in soils at low concentrations. However, mines, foundries, and smelters, are the main sources that are often associated with high levels of contamination in soils (Alloway and Alloway, 2013). Plants growing on contaminated soils may accumulate heavy metals in their edible parts, which and can result in severe health risks for foraging animals and humans if they enter the food chain (Peralta-Videa et al., 2009; Khan et al., 2015). Heavy metals can disrupt basic metabolic processes, cause displacement of essential metals from biomolecules, and lead to the formation of reactive oxygen species (ROS). Mercury (Hg) is considered the most toxic heavy metal, and Hg contamination is increasing worldwide in both agricultural soils and wild ecosystems. Environmental Hg pollution can be due to natural processes (soil erosion, volcanism, wild fires, and geothermal activities), but is mostly the result of the above-mentioned anthropogenic activities. Bioavailable Hg is readily taken up from the soil and water by different organisms, thus entering the food chain and leading to serious toxicity problems. Mercury has major adverse effects on plants, it creates osmotic stress by competing with necessary nutrients for absorption from the soil, in turn producing a disruption of the plasma membrane. Once inside the cells, Hg has an affinity for sulfhydryl groups and it is capable of binding to enzymes and proteins, replacing essential ions, thereby preventing them from playing their role in the different cellular pathways. In addition, its great capacity to produce cell damage is the formation and accumulation of ROS: superoxides, hydrogen peroxides and hydroxyl radicals (Patra and Sharma, 2000). The molecular mechanisms involved in Hg toxicity and tolerance in plants have been widely investigated (Ortega-Villasante et al., 2005, 2007; Zhou et al., 2007, 2008). A complex cellular defense system involves both enzymatic and non-enzymatic factors in ROS scavenging (Gill and Tuteja, 2010). Antioxidant enzymes superoxide dismutase, catalase ascorbate peroxidase, guaiacol peroxidase, and glutathione-S-transferases constitute the first defense. The non-enzymatic response regulates the production of phytochelatins, polyamines, and metallothioneins, as well as low-molecular weight compounds, such as reduced ascorbic acid, proline, α-tocopherol, glutathione, and different secondary metabolites, including phenolics, carotenoids, and flavonoids (Akram et al., 2017).

Flavonoids make up a very broad group of polyphenolic compounds formed by two aromatic rings (A and B) linked by three carbon atoms that can form another ring C (Panche et al., 2016). They are capable of scavenging ROS because they have hydroxyl groups either in ring A or ring B, which react easily with the free oxygen radicals (Mierziak et al., 2014; Panche et al., 2016). Beside scavenging metal-induced ROS, flavonoids may detoxify metal(oid)s by ion chelation followed by vacuolar sequestration (Ahammed and Yang, 2022). Certain flavonoids are also involved in legume nodule initiation, as reported for a flavone (7,4′-dihydroxyflavone) and a flavonol (kaempferol) in *Medicago truncatula* (Zhang et al., 2009). Flavonoids are classified into different families that include chalcones, flavanols, flavanones, flavones, flavonols, anthocyanidins, isoflavonoids, and neoflavonoids. This structural variation is combined with a wide variety of decorations that include acylations, hydroxylations, methoxylations, prenylations, or glycosylations (Dixon and Pasinetti, 2010). Glycosides are the most common form of flavonoid derivatives, frequently O-glycosides, and less frequently, C-glycosydes (Rauter et al., 2019). Glycosylation may occur with one or multiple sugars units linked to the aglycone moiety in different positions (Harborne and Baxter, 1999). UDP-glycosyltransferases (UGTs) constitute a wide enzyme family and able to glycosylate flavonoids, as well as many other metabolites (Behr et al., 2020). Sugar substitution might modify their antioxidant capacity (Zheng et al., 2017), but also their cellular and tissular location (Taguchi et al., 2000) and it might retroactively regulate their biosynthetic pathways (Zhang and Liu, 2014). Flavonoid aglycones are potentially more antioxidant than their glycosylated forms (Chae et al., 2006), while quercetin and kaempferol glycosylation results in higher ROS scavenging activity (Zhao et al., 2019). Glycosylation of anthocyanins leads to their storage in the vacuole, thus supporting the biosynthesis of these flavonoids *via* feedback inhibition (Li et al., 2017).

Legumes are capable of establishing a symbiotic relationship with certain soil bacteria, collectively known as rhizobia, that leads to the formation of the root nodule, a new organ where bacteria are able to fix atmospheric nitrogen. This symbiotic interaction allows legumes to act as colonizers of degraded soils with little organic matter (Coba de la Peña and Pueyo, 2012). The Rhizobium-legume symbiosis is also considered a potentially powerful tool in the reclamation of polluted soils, due to the pioneer characteristics of legumes and the beneficial effects of the bacteria, which besides making nitrogen fixation possible, have their own detoxifying mechanisms that favor the establishment of the plants (Arregui et al., 2021). Tolerance to stress in legumes needs to consider the symbiotic system, the plant and the bacteria, and can be achieved by cultivar and inoculant selection or by transgenic approaches (Coba de la Peña and Pueyo, 2012). *Medicago truncatula* is a forage legume with high biomass and soil coverage that may represent a suitable pioneer plant in metal-polluted soils, provided that tolerant cultivars are identified, as compatible tolerant rhizobia have been isolated from Hg-contaminated soils (Nonnoi et al., 2012). Moreover, high quality reference genomes and gene annotations (Young et al., 2011; Tang et al., 2014), a HapMap panel of resequenced germplasm (Stanton-Geddes et al., 2013), and a large mutant collection (Lee et al., 2018) are available for this model legume.

In previous studies we were able to identify Hg-tolerant and Hg-sensitive *M. truncatula* cultivars (García de la Torre et al., 2013; Paape et al., 2022) by germplasm screening. As indicated above, the general molecular mechanisms of Hg tolerance and response in plants have been described; however, to our knowledge, the role of flavonoids in the response to Hg stress, has not been investigated. In the present work we aimed to identify the flavonoid profile of Hg-tolerant and Hg-sensitive *M. truncatula* cultivars and to investigate whether the metabolomic response to Hg stress was related to the level of Hg tolerance. All main subclasses of plant flavonoids are present in the genus *Medicago* (Gholami et al., 2014). Additionally, in a recent genome-wide association study (GWAS) on Hg tolerance in *M. truncatula* (Paape et al., 2022), we identified a UGT gene as a candidate gene within the proximity to the top SNP related to Hg tolerance. Several additional UGT genes were also present in the same region of chromosome 2, and more than 150 UGTs have been identified in the *M. truncatula* genome (Modolo et al., 2007). While the specific substrates of the UGTs nearby the significant SNP are unknown, it is of interest to analyze here the possible role of flavonoid glycosylation in response to Hg stress in *M. truncatula*.

## Materials and Methods

### Plant Material, Plant Growth, and Mercury Treatment

Three Hg-tolerant and one Hg-sensitive cultivar within the HapMap panel were selected according to their seedling relative root growth (RRG), which represents a valid indicator of Hg tolerance in *M. truncatula* (García de la Torre et al., 2013). Tolerant accessions HM081 (RRG = 99.39), HM080 (RRG = 92.00), and HM175 (RRG = 89.96), and sensitive cultivar HM289 (RRG = 21.74) were selected (Paape et al., 2022). Here we will refer to cultivars HM081, HM080, HM175, and HM289 as LR1, LR2, LR3, and LR4, respectively, with L indicating leaves and R indicating roots.

Seeds were obtained from the University of Minnesota, *Medicago* HapMap project (medicagohapmap2.org/germplasm). Seed were scarified with 96% sulfuric acid for 6 min, washed six times with sterile water, further sterilized for 1 min in commercial bleach and washed six times with sterile water, and imbibed in sterile water for 1 h. Seed were germinated in Petri dishes containing agar-water (10 g L^–1^) for 48 h in the dark at 25°C. Germinated seedlings were inoculated with Hg-tolerant *Ensifer medicae* AMp08 (Nonnoi et al., 2012) by immersion in a fresh bacterial culture (OD_600 nm_ = 0.8) for 30 min. Seedlings were then transferred to pots (6 cm × 6 cm × 8 cm) containing sterile vermiculite and watered with nitrogen-limiting Hoagland nutrient solution [2.6 mg L^–1^ KNO_3_, 0.68 g L^–1^ KH_2_PO_4_, 0.182 g L^–1^ CaCl_2_⋅2H_2_O, 0.615 g L^–1^ MgSO_4_⋅7H_2_O, 0.109 g L^–1^ K_2_SO_4_, 0.205 g L^–1^ Hampiron (Rhône Poulenc), and 1.35 mL of a solution containing 11 g L^–1^ H_3_BO_3_, 6.2 g L^–1^ MnSO_4_⋅H_2_O, 10 g L^–1^ KCl, 1 g L^–1^ ZnSO_4_⋅7H2O, 1 g L^–1^ (NH_4_)_6_Mo_7_O_24_⋅4H_2_O, 0.5 g L^–1^ CuSO_4_⋅5H_2_O and 0.5 mL L^–1^ H_2_SO_4_]. Plants were grown under controlled conditions (180 μmol photon m^–2^ s^–1^, 25/20°C, 16/8 h photoperiod, 65% relative humidity) for 6 weeks. One set of plants was then treated with mercury using 300 mM HgCl_2_ added to the nutrient solution. This treatment did not produce any apparent changes in growth parameters, but led to a significant reduction of nitrogenase activity, which was ∼70 % of that of control plants. A second set of plants was given no heavy metal treatment and was used as a control group. After 3 days of treatment, roots and leaves were collected separately in Eppendorf tubes containing three sterile 3-mm AISi 304 steel balls, and tissues were homogenized using a MM400 mixer mill (RETSCH, Haan, Germany). The samples were shaken at a frequency of 30 s^–1^ in six periods of 30 s. To avoid deterioration of the samples during the process, they were immersed in liquid nitrogen between each period. Homogenized samples were frozen in liquid nitrogen and stored at –80°C.

### Flavonoid Extraction

The crushed material, approximately 1 g, of leaves and roots of the four target varieties: LR1, LR2, LR3, and LR4, was kept for 12 h in a lyophilizer at a pressure of 0.05 mbar to remove all the moisture. A methanol-water extraction buffer (70–30%) was used, with which a higher concentration of phenolic compounds dissolved in the buffer is achieved (Muñoz et al., 2015). One mL of the extraction buffer was added to 25 mg of lyophilized material reaching a concentration of 25 mg mL^–1^ and placed in the sonicator for half an hour. It was centrifuged for 15 min at 14,800 rpm and 4°C, and the supernatant obtained (containing the extracted flavonoids) was distributed in UHPLC vials.

### Liquid Chromatography-Tandem Mass Spectrometry (UPLC- Q-TOF-MS/MS)

An Agilent 1290 UHPLC system coupled to an Agilent 6540 quadrupole time-of-flight mass spectrometer (q-TOF MS) equipped with an orthogonal ESI source was employed for the phytochemical profiling of *M. truncatula* leaves and roots extracts. Chromatographic separation was conducted using a Zorbax Eclipse Plus C18 column (2.1 mm × 100 mm, 1.8 μm particle diameter, Agilent Technologies, Santa Clara, CA, United States) at 30°C. The mobile phase was composed of water (0.1% formic acid, solvent A) and acetonitrile (0.1% formic acid, solvent B). A 5 μL aliquot of the sample was injected at a flow rate of 0.5 mL/min during gradient elution. The gradient program was as follows: 0 min, 0% B; 7 min, 30% B; 9 min, 80% B; 11 min, 100% B; 13 min, 100% B; 14 min, 0% B. The mass spectrometer was operated in MS and MS/MS modes for the structural analysis of all compounds. MS parameters were the following: capillary voltage, 4,000 V; nebulizer pressure, 40 psi; drying gas flow rate, 10 L/min; gas temperature, 350°C; skimmer voltage, 45 V; fragmentor voltage, 110 V. The MS and Auto MS/MS modes were set to acquire m/z values ranging between 50–1,100 and 50–800, respectively, at a scan rate of 5 spectra per second.

A randomized sequence of samples, including three replicates of each control and treated sample, was analyzed in triplicate. An additional pool of all samples, combining equal aliquots from each sample, was injected regularly throughout the samples sequence, as quality control, to monitor the stability of the analysis. A mix solution of 10 standards, were injected regularly throughout the samples sequence, as second quality control, to validate the LC-HRMS method in terms of retention time and signal variability, and mass accuracy.

### Flavonoid Metabolomics Data Analysis

Agilent Mass Hunter Qualitative analysis software version B.07.00 and Agilent Mass Hunter Quantitative (for Q-TOF) analysis software version B.08.00 were used for post-acquisition data processing. The Molecular Formula Generator algorithm within the Agilent Mass Hunter software was also to enhance the metabolite database search and mass accuracy calculation (<10 ppm). The accurate mass data, ion source fragmentation, MS/MS fragmentation patterns, MS databases (i.e., NIST, METLIN, and HMDB) and bibliographic search were employed for tentative identification of the phenolic compounds present in the samples.

Both control and Hg-treated samples were submitted to statistical analysis using the online MetaboAnalyst program^1^. Univariate analysis based on *T*-test and Fold change (FC) analysis was applied to detect differentially accumulated flavonoids (*p*-value cut-off: 0.05) with a defined absolute value of change (FC > 2.5) between control and treated samples. To improve data interpretation, multivariate data analysis based on a cluster heatmap hierarchical clustering and principal component analysis (PCA) was carried out. Hierarchical clustering was applied using a complete linkage clustering method with Pearson distance measurement. PCA was carried out using the statistical software The Unscrambler V9.7 (CAMO Software AS, Oslo, Norway). Multivariate data matrix was analyzed after data autoscaling (data were mean-centered and divided by the standard deviation of each variable).

## Results

### Flavonoids Metabolomics Analysis of *Medicago truncatula* Cultivars With Different Degree of Hg Tolerance

Leaves and roots of the selected *M. truncatula* varieties grown under control conditions were subjected to an extensive flavonoid profiling analysis to evaluate their accumulation profiles. Full-scan HRMS data obtained in ESI(+) ionization mode were screened for expected polyphenols reported in *M. truncatula* (Jasiński et al., 2009; Marczak et al., 2010; Staszków et al., 2011; Gholami et al., 2014). This targeted approach allowed chromatographic peak detection of suspected flavonoids, on the basis of accurate mass and isotopic distribution. Further structural information was obtained from HRMS/MS data acquired by data-dependent scan in auto MS/MS mode. Diagnostic product ions and neutral loss filtering of MSMS data were applied to screen for structurally related flavonoids, including isomers and glycoconjugate derivatives with different substitution patterns on the aglycone.

Following the proposed profiling strategy, the analyzed extracts of *M. truncatula* revealed the presence of at least 46 phenolic compounds, classified into five different flavonoid families: three anthocyanidins (**1, 12, 21**), 26 flavones (**2, 4–10, 13–16, 18–20, 22–28, 30, 34, 35, 40**), 14 isoflavones (**3, 11, 17, 29, 31–33, 38, 39, 42–46**), two pterocarpan flavonoids (**37, 41**), and one flavanone (**36**; Table 1).

**TABLE 1:** Tentatively identified flavonoids and their glycoconjugates from *Medicago truncatula* roots and leaves by ESI(+)-q-TOF-MS/MS analysis, including the retention time (min), molecular formula, experimental molecular ions, calculated mass error (Δm/z, ppm), and MS/MS product ions.

Peak no	Ret. time (min)	Family	Key	Tentative identification	Formula	Monoisotopic mass	m/z [M+H]+ (theoretical)	Error (ppm)	MS/MS product ions (m/z)
1	4.60	Anthocyanidin	Peon-G01	Peonidin Glc I	C22H23O11+	463.1240	463.1240	1.8	301.0702
2	4.61	Flavone	Tric-G01	Tricin GlcGlcA I	C29H32O18	668.1589	669.1662	0.3	507.1119, 331.0817
3	5.07	isoFlavone	Afror-G01	Afrormosin Glc I	C23H24O10	460.1369	461.1442	–0.4	461.1418, 299.0905
4	5.13	Flavone	Tric-G02	Tricin GlcGlcA II	C29H32O18	668.1584	669.1657	–0.2	507.1119, 331.0817
5	5.18	Flavone	Api-G01	Apigenin GlcAGlcA	C27H26O17	622.1170	623.1243	–0.2	271.0592
6	5.39	Flavone	Chry-G01	Chrysoeriol GlcAGlcA	C28H28O18	652.1280	653.1353	0.9	477.1025, 301.0709
7	5.49	Flavone	Tric-G03	Tricin GlcAGlcA	C29H30O19	682.1387	683.1460	1.4	507.1119, 331.0817
8	5.77	Flavone	Tric-G04	Tricin GlcGlcA III	C29H32O18	668.1590	669.1663	0.0	507.1119, 331.0817
9	5.81	Flavone	Api-G02	Apigenin FerGlcAGlcAGlcA I	C43H42O26	974.1975	975.2048	–0.2	799.1716, 447.0922, 323.0769, 271.0598
10	5.90	Flavone	Api-G04	Apigenin CouGlcAGlcAGlcA I	C42H40O25	944.1865	945.1938	–0.2	769.1589, 447.0922, 323.0769, 271.0598
11	5.93	isoFlavone	Form-G01	Formononetin Glc I	C22H22O9	430.1264	431.1337	0.9	269.0798
12	5.96	Anthocyanidin	Peon-G02	Peonidin Glc II	C22H23O11+	463.1240	463.1240	1.8	301.0702
13	5.97	Flavone	Chry-G02	Chrysoeriol FerGlcAGlcAGlcA	C44H44O27	1,004.2073	1,005.2146	–0.1	477.1025, 301.0709
14	6.03	Flavone	Tric-G05	Tricin Glc I	C23H24O12	492.1268	493.1341	–2.1	331.0817
15	6.08	Flavone	Api-G05	Apigenin FerGlcAGlcAGlcA II	C43H42O26	974.1970	975.2043	–0.2	799.1716, 447.0922, 323.0769, 271.0598
16	6.19	Flavone	Api-G03	Apigenin GlcA	C21H18O11	446.0849	447.0922	–0.9	271.0587
17	6.29	isoFlavone	Bioch-G01	Biochanin A MalGlc I	C25H24O13	532.1217	533.1290	1.8	285.0762
18	6.30	Flavone	Api-G07	Apigenin CouGlcAGlcAGlcA II	C42H40O25	944.1865	945.1938	–0.2	769.1589, 447.0922, 323.0769, 271.0598
19	6.32	Flavone	Api-G06	Apigenin FerGlcAGlcA I	C37H34O20	798.1643	799.1716	–1.1	447.0926, 271.0601, 353.0876
20	6.36	Flavone	Api-G08	Apigenin CouGlcAGlcA I	C36H32O19	768.1538	769.1611	–0.8	447.0922, 323.0769, 271.0598
21	6.40	Anthocyanidin	Peon-G03	Peonidin Glc III	C22H23O11+	463.1240	463.1240	1.8	301.0702
22	6.40	Flavone	Api-G09	Apigenin FerGlcAGlcAGlcA III	C43H42O26	974.1965	975.2038	0.1	799.1716, 447.0922, 323.0769, 271.0598
23	6.41	Flavone	Chry-G03	Chrysoeriol GlcA	C22H20O12	476.0955	477.1028	–1.5	477.1025, 353.0553, 301.0709
24	6.48	Flavone	Tric-G06	Tricin Glc II	C23H24O12	492.1268	493.1341	–2.1	331.0817
25	6.53	Flavone	Tric-G07	Tricin FerGlcAGlcA I	C39H38O22	858.1855	859.1928	–0.3	507.1119, 331.0817
26	6.58	Flavone	Api-G10	Apigenin FerGlcAGlcA II	C37H34O20	798.1643	799.1716	–0.6	447.0920, 271.0607, 353.0876
27	6.61	Flavone	Tric-G08	Tricin CouGlcAGlcA I	C38H36O21	828.1727	829.1800	–0.6	507.1119, 331.0817
28	6.72	Flavone	Tric-G09	Tricin Glc III	C23H24O12	492.1268	493.1341	–2.1	331.0817
29	6.83	isoFlavone	Form-G02	Formononetin Glc II	C22H22O9	430.1264	431.1337	0.9	269.0798
30	6.92	Flavone	Api-G11	Apigenin CouGlcAGlcA II	C36H32O19	768.1538	769.1611	–0.9	447.0922, 323.0769, 271.0598
31	6.94	isoFlavone	Daiz	Daidzein	C15H10O4	254.0579	255.0652	–1.6	255.0649
32	6.95	isoFlavone	Bioch-G02	Biochanin A MalGlc II	C25H24O13	532.1217	533.1290	0.5	285.0762
33	6.97	isoFlavone	Afror-G02	Afrormosin Glc II	C23H24O10	460.1369	461.1442	–2.8	461.1418, 299.0905
34	7.06	Flavone	Tric-G10	Tricin CouGlcAGlcA II	C38H36O21	828.1727	829.1800	–2.8	507.1119, 331.0817
35	7.19	Flavone	Tric-G11	Tricin FerGlcAGlcA III	C39H38O22	858.1855	859.1928	0.8	507.1119, 331.0817
36	7.29	Flavanone	Narin-G	Naringenin Chalcone Glc	C21H22O10	434.1213	435.1286	–0.7	391.1364, 271.0432
37	7.60	Pterocarpan	Medi-G01	Medicarpin MalGlc I	C25H26O12	518.1424	519.1497	–1.0	271.0964
38	7.67	isoFlavone	Form-G03	Formononetin MalGlc	C25H24O12	516.1268	517.1341	–1.5	269.0798
39	7.71	isoFlavone	Afror-G03	Afrormosin MalGlc	C26H26O13	546.1373	547.1446	–0.4	547.1444, 299.0907
40	8.07	Flavone	Tric-G12	Tricin Glc IV	C23H24O12	492.1268	493.1341	–2.1	331.0817
41	8.15	Pterocarpan	Medi-G02	Medicarpin MalGlc II	C25H26O12	518.1424	519.1497	–1.0	271.0964
42	8.24	isoFlavone	BiochA	Biochanin A	C16H12O5	284.0684	285.0757	–1.4	285.0757
43	8.39	isoFlavone	Iriso-Iso	Irisolidone isomer	C17H14O6	314.0790	315.0863	–3.4	315.0863
44	8.56	isoFlavone	Form	Formononetin	C16H12O4	268.0735	269.0808	–2.6	269.0806
45	8.64	isoFlavone	Afror	Afrormosin	C17H12O5	298.0841	299.0914	–1.3	299.0902
46	9.11	isoFlavone	Iriso	Irisolidone	C17H14O6	314.0790	315.0863	–3.1	315.0863

*Abbreviations of sugars and acyl groups: GlcA, glucuronic acid; Glc, glucose; Mal, malonic acid; Fer, ferrulic acid; and Cou, Coumaric acid.*

**FIGURE 1:**
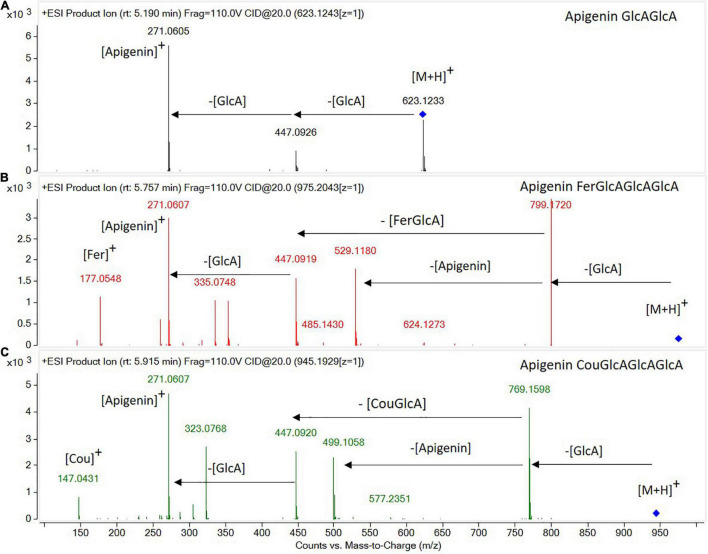
High-resolution tandem mass spectrometry spectra obtained in the ESI(+)-QTOF-MS/MS mode for the identification of flavone glycoconjugates: **(A)** apigenin diglucuronide, **(B)** apigenin feruloyl triglucuronide, and **(C)** apigenin coumaroyl triglucuronide.

Most of the compounds detected were conjugated flavonoids, derivatives of apigenin, tricin, chrysoeriol, biochanin, afrormosin, formonentin, and peonidin, mainly glycosylated with glucose and/or glucuronic acid molecules. Some of these compounds were also acylated with ferulic, coumaric, or malonic acid. Sugar moieties (e.g., GlcA-glucuronic acid, Glc-glucose) and acyl groups on sugar rings (e.g., Fer-ferrulic acid, Cou-Coumaric acid, Mal-malonic acid) were annotated based on characteristic neutral losses and product ions (Figure 1). Thus, the CID-MS/MS (collision-induced dissociation tandem mass spectrometry) pattern of flavonoid glycosides was mainly characterized by the cleavages of consecutive glycosidic bonds, either between glucoside or glucuronic acid molecules, or between sugar and the flavone moieties (Figure 1A). However, the CID-MS/MS spectra of acylated glycoconjugates showed product ions with charge retained on glucuronic acid moieties acylated with ferulic (Figure 1B) or coumaric acid (Figure 1C).

**FIGURE 2:**
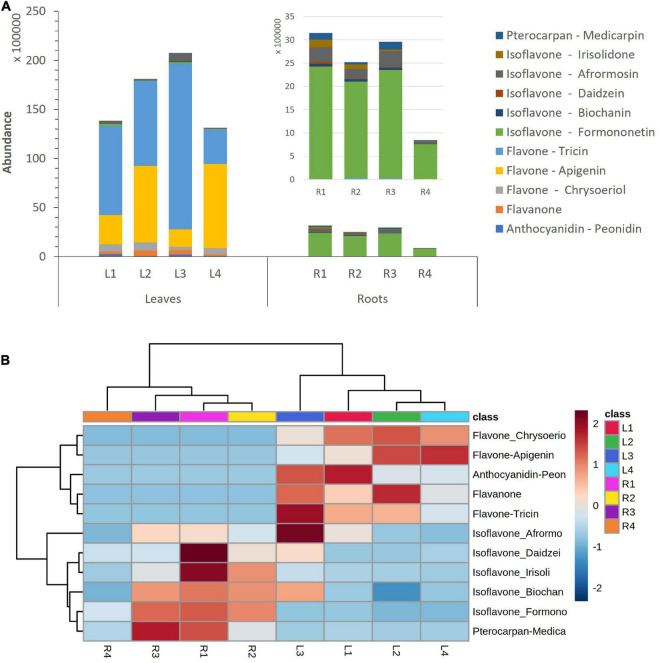
**(A)** Accumulation of flavonoid families in *Medicago truncatula* leaves and roots. **(B)** Dendrogram and heatmap of flavonoid families in *M. truncatula* leaves (L) and roots (R).

Substantial differences in flavonoid accumulation were observed among leaves and roots for the studied *M. truncatula* varieties. The abundance of total flavonoids in leave samples was remarkably higher than in roots; accounting for a total content between 4.6- and 16.0-fold higher (Figure 2A). Thus, an increase in flavonoid’s accumulation ratio between leaves and roots could be observed: L/R1 (4.6) < L/R2 (7.0) < L/R3 (7.2) << L/R4 (16), as Hg-tolerance decreases (LR1 > LR2 > LR3 >> LR4). Figure 2B displays the relative abundance of different flavonoid families in *M. truncatula* leaves and roots for the target varieties. The heatmap reveals higher levels of tricin-, apigenin-, and chrysoeriol-type flavones, as well as peonidin-type anthocyanidins in leaf samples, whereas higher concentrations of daidzein-, biochanin A-, irisolidone-, and formononetin-type isoflavones were found in roots.

**FIGURE 3:**
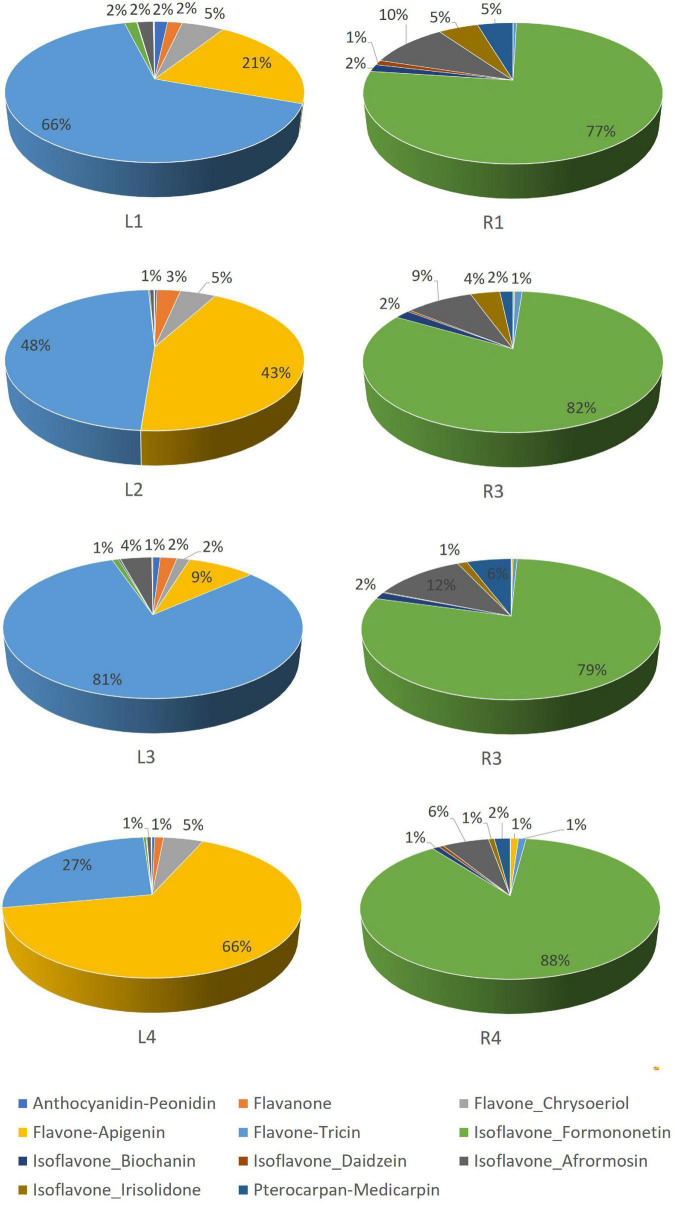
Relative abundance (%) of flavonoid families in *Medicago truncatula* leaves and roots.

Leaves contained mainly tricin and apigenin glycoconjugates, accounting for a total contribution ranging from 81 to 93% of total flavonoids (Figure 3 and Supplementary Table 1). Hg-tolerant variables (LR1, LR2, and LR3) accumulate tricin-type flavones (48–81%) as major compounds, whereas the most Hg-sensitive variable (LR4) accumulates mainly apigenin-type flavones (66%) in leave samples. Unlike the aerial part, roots contained mainly formononetin-type isoflavones (77–88%).

**FIGURE 4:**
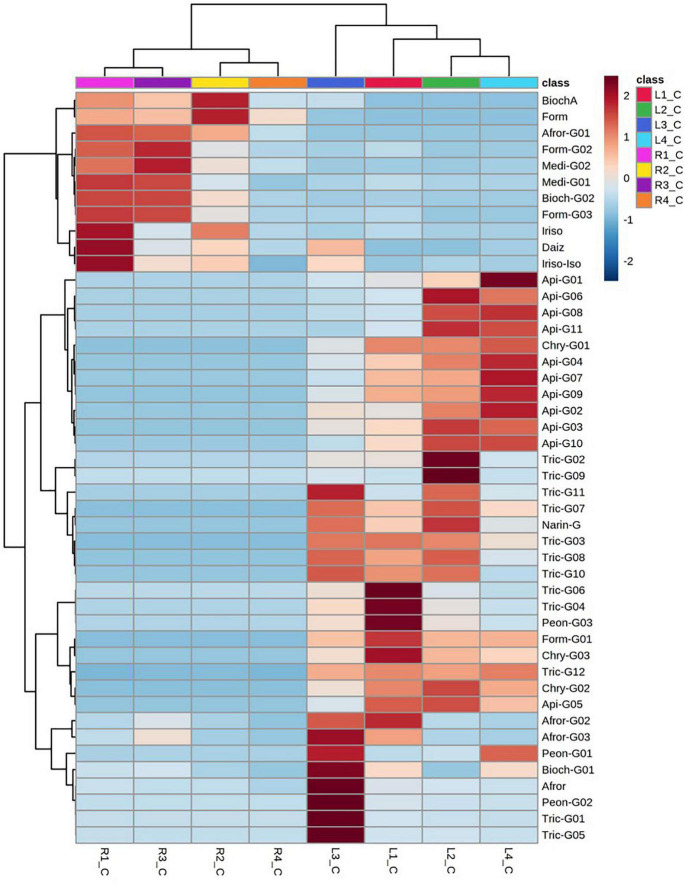
Dendrogram and heatmap of the flavonoids and their glycoconjugates in *Medicago truncatula* leaves (-L) and roots (-R).

**FIGURE 5:**
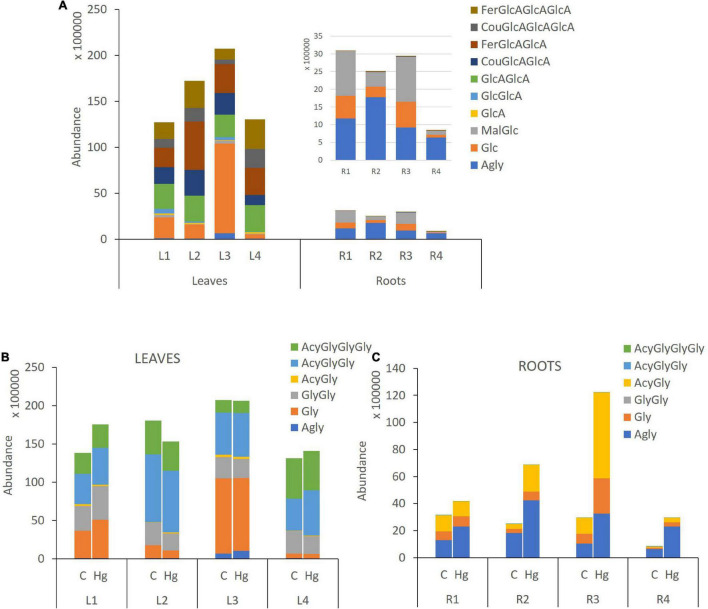
Flavonoid glycosylation profile in leaves and roots of *Medicago truncatula*. **(A)** Control samples, **(B)** Control vs Hg-treated varieties of leaves, and **(C)** Control vs Hg-treated varieties of roots. Agly, aglicone; GlcA, glucuronic acid; Glc, glucose; Mal, malonic acid; Fer, ferrulic acid; Cou, Coumaric acid; Gly, monoglycosylated; GlyGly, diglycosylated; GlyGlyGly, triglycosylated; and Acy, acylated.

A comparative flavonoid profiling analysis of the target varieties can be observed in the heatmap of Figure 4, showing a color code from high to low concentration ranging from dark red to dark blue. Leaves and root samples were grouped according to their flavonoid content based on a clusters analysis (Figure 4). Most of flavonoids detected in leaves are glycoconjugated derivatives containing from 1 to 3 sugar units (glucose or glucuronic acid; Figure 5A). The accumulation levels of free aglycones in leaves is below 3% of the total flavonoids content. Apigenin mono- and diglucuronide (**16, 5**), as well as a set of positional isomers of apigenin di- and triglucuronides acylated with ferrulic (**9, 15, 19, 22, 26**) or coumaric (**10, 18, 20, 30**) acids are highly abundant in LR4, followed by LR2, LR1, and LR3. Another major group of flavones in leave samples involve tricin monoglucosides (**14, 24, 28, 40**), glucuronyl-glucosides (**2, 4, 8**), diglucuronides (**7**), feruloyl-diglucuronides (**25, 35**), and coumaroyl-diglucuronides (**27, 34**). The accumulation of these tricin glycosides is higher in Hg-resistant varieties than in the Hg-sensitive cultivar. Wider accumulation variability for the different varieties was observed for chrysoeriol mono- and diglucuronide (**23, 6**), chrysoeriol feruloyl-triglucuronide (**13**) and the three positional isomers of the anthocyanidin peonidin glucoside (**1, 12, 21**).

Flavonoid aglycones are considered more antioxidant than their glycosylated forms (Chae et al., 2006). The detected levels of free aglycones in roots are between 36 and 77%, depending on the variety (Figure 5A), and the conjugated derivatives were mainly monoglucosylated and malonylated flavonoids. Free isoflavones such as biochanin A (**42**), formononetin (**44**), irisolidone (**43, 46**), and daidzein (**31**) were highly enriched in root samples. The levels of glucosylated and malonylglucosylated derivatives of medicarpin (**37, 41**) and its isoflavone precursors formononetin glucoside and malonylglucoside (**29, 38**), as well afrormosin glucoside (**3**) and biochanin A malonylglucoside (**32**), were mainly present in Hg-tolerant root samples. According to the flavonoid profiling analysis carried out in control samples of leaves and root tissues, the synthesis of free isoflavones, followed by monoglycosylation and further malonylation was shown to be more characteristic of roots samples, whereas a higher glycosylation degree (di-and triglycosides), followed by further acylation with coumaric and ferulic acid were more characteristic of leave tissues.

### Differential Flavonoid Accumulation in *Medicago truncatula* Cultivars in Response to Hg Stress

**FIGURE 6:**
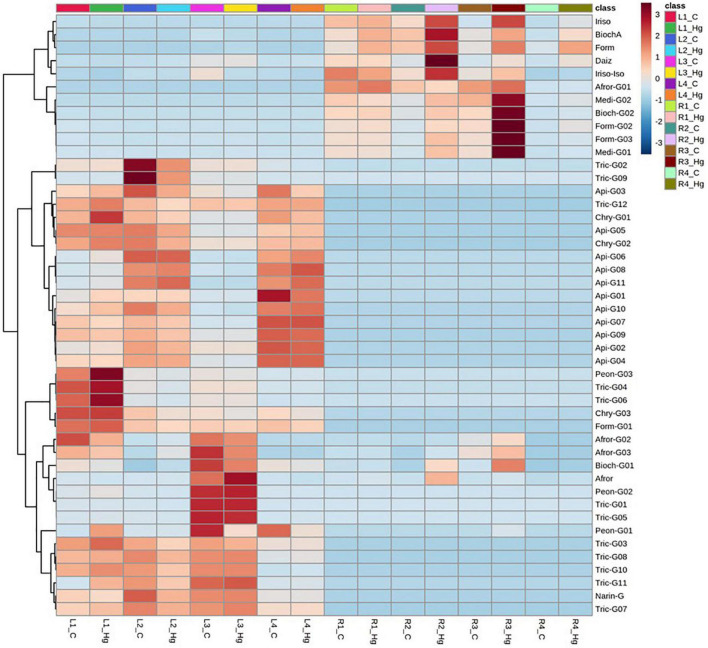
Dendrogram and heatmap analysis of flavonoids and their glycoconjugates profiling in *Medicago truncatula* leaves (-L) and roots (-R) for control (C) and Hg-treated plants.

Despite the observed differences in flavonoid profiles among the target *M. truncatula* varieties and among leaves and roots samples, the resulted obtained after Hg-treatment suggest flavonoids accumulation changes of different magnitude, according to the tissue sample (leaves or roots). Total flavonoids accumulation and glycosylation profiles for control and Hg-treated samples are comparatively displayed in Figures 5B,C, whereas the effects of Hg stress on individual flavonoids profile are shown in Figure 6. A set of differentially expressed metabolites, exhibiting accumulation values with FC > 2.5 (upregulated: Log_2_ FC > 1.3) or FC < 0.4 (downregulated: Log_2_ FC < –1.3) in Hg-treated compared to control samples, is summarized in Table 2.

**TABLE 2:** Differentially accumulated flavonoids (Log_2_ FC) in *Medicago truncatula* varieties after Hg-treatment.

COMPOUND NAME	LEAVES				ROOTS			
	L1	L2	L3	L4	R1	R2	R3	R4
Afrormosin						3.3		2.3
Afrormosin Glc I								3.3
Afrormosin MalGlc		1.4				1.6		
Apigenin GlcAGlcA					2.4			
Apigenin FerGlcAGlcAGlcA I						3.7	1.6	
Apigenin CouGlcAGlcAGlcA I						3.6	3.8	
Apigenin FerGlcAGlcAGlcA II								2.1
Biochanin A MalGlc I		.5				2.7	2.2	2.2
Biochanin A MalGlc II	2.1					1.7	2.1	1.8
Biochanin A						1.3	1.4	1.9
Daidzein	2.6	1.9				3.4	1.6	2.3
Formononetin			2.1				1.6	1.8
Formononetin Glc II	1.4	2.4					2.3	2.6
Formononetin MalGlc	1.4	3.3				2.4	2.5	1.8
Irisolidone						1.6	3.7	2.9
Irisolidone isomer	1.6		1.6			1.6		1.3
Medicarpin MalGlc I						2.5	2.4	1.9
Medicarpin MalGlc II	2.6		2.4	4.1		1.5		
Peonidin Glc I	2.9		1.8	1.6				
Tricin GlcAGlcA						3.3		1.4
Tricin Glc I							3.1	
Tricin FerGlcAGlcA I						3.1		
Tricin CouGlcAGlcA I					3.1	4.8		
Tricin FerGlcAGlcA III	2.2							

*Upregulated: Log_2_FC > 1.33 (red); Downregulated: Log_2_FC < 1.33 (green).*

*
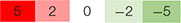
*

Total flavonoid accumulation results in leave samples (Figure 5B) showed slight changes in some varieties (L1 and L2) after they were Hg-treated, although in general no significant variations were observed either in the flavonoid profile or in the glycosylation degree. Comparing the expression profile of individual flavonoids in control and Hg-treated varieties (Figure 6), similar flavonoid accumulation patterns could be observed in general, except for some particular metabolites. Thus, the monoglycosylated and malonylated derivatives of peonidin (Peon-G01), medicarpin (Medi-G02), were strongly deregulated metabolites in L1, L3, and L4 varieties, whereas malonylglucosides of biochanin A (Bioch-G02 and Bioch-G01) and formononetin (Form-G02 and Form-G03) were mainly deregulated in L1 an L2 under Hg stress. This behavior suggests that treatment with Hg does not cause generalized alterations with respect to the basal state, but rather punctual alterations in the synthesis of specific flavonoids in leaves.

Unlike leaf samples, significant quantitative changes could be observed in the accumulation of a wider range of flavonoids and their conjugate derivatives in roots, as a result of the Hg treatment in. As displayed in Figure 5C, the stress caused by Hg caused a significant increase in both the levels of free and glycosylated flavonoids. The most abundant aglycone in roots, formononetin, exhibited increasing FC values: R1 (1.8) > R2 (2.3) > R3 (3.1) > R4 (3.6), as Hg-tolerance decreases. A similar trend was shown for formononetin monoglycoside R1 (1.1) > R2 (2.1) > R3 (3.6) > R4 (3.8). The expression profile of individual flavonoids in roots under Hg stress was notably more altered compared to leave flavonoids profile under the same conditions, as clearly observed in Figure 6. Eight flavonoids were strongly upregulated in three of the target root varieties, involving malonylglucosides of biochanin A (Bioch-G01, Bioch-G02), formononetin (Form-G03), and medicarpin (Medi-G01), followed by the aglycones biochanin, daidzein, and irisolidone.

**FIGURE 7:**
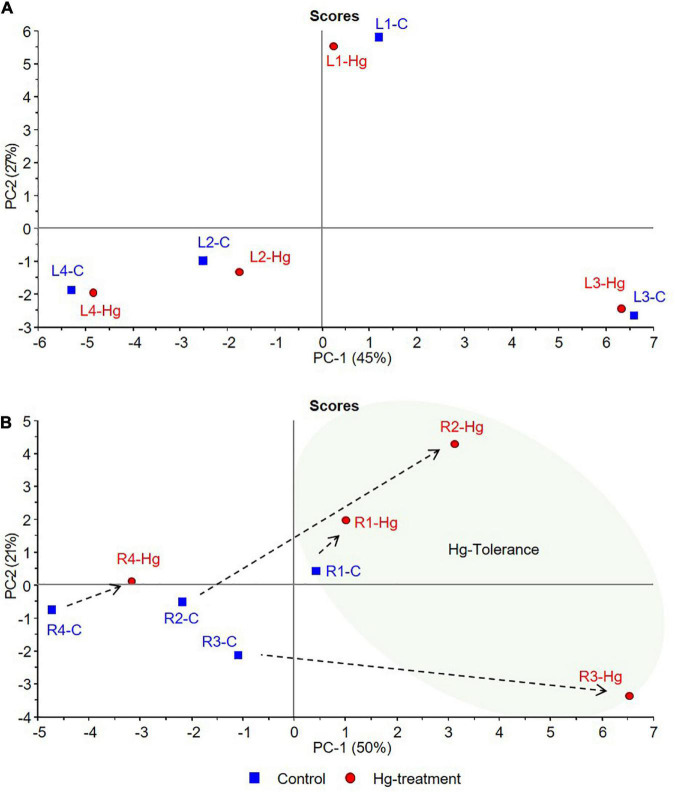
PCA score plots of control (C, blue) and Hg-treated varieties (Hg, red) of *Medicago truncatula*, based on individual flavonoid accumulation. **(A)** Leaves and **(B)** Roots.

**FIGURE 8:**
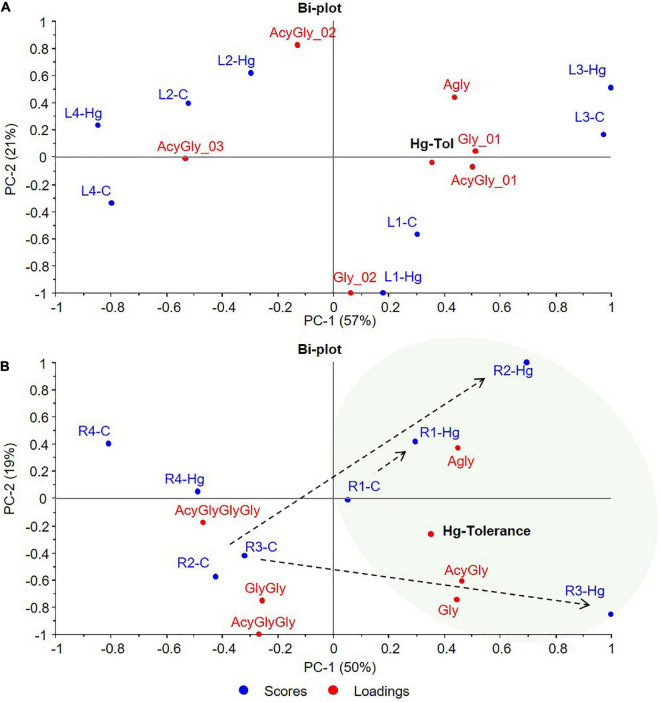
PCA bi-plots of control (C, blue) and Hg-treated varieties (Hg, red) of *Medicago truncatula*, based on the flavonoids glycosylation degree. **(A)** Leaves and **(B)** Roots. Agly, aglycone; Gly, monoglycosylated; GlyGly, diglycosylated; GlyGlyGly, triglycosylated; and Acy, acylated.

The results presented above evidence the alteration of the flavonoids metabolic profile and their glycosylation processes in response to Hg stress. However, notable differences have been seen between varieties, both in the basal metabolic profile and in the response to treatment with Hg. In order to explore patterns of metabolic response (flavonoid accumulation) to Hg stress as a function of the tolerance of each variety, a multivariate data analysis was carried out. For this purpose, flavonoid compounds identified in the targeted profiling analysis along with the Hg-tolerance values obtained for each variety of *M. truncatula* were jointly evaluated through a PCA. The proposed unsupervised multivariate analysis tool allows the evaluation of the compositional variability of the data in order to obtain the correlation structure between flavonoids accumulation and Hg tolerance values for the target varieties. Associations between variables were established by proximity in the multivariate space. Two PCA were carried out to evaluate the possible associations between individual flavonoids (Figure 7), and flavonoid glycosylation degree (Figure 8), with the Hg-tolerance of the studied varieties.

According to the PCA results, graphically displayed in Figure 7, the first two principal components (PC1, PC2) explained the 72 and 71% of the total variance for leaves and roots, respectively. In both tissue samples, a trend from lower to higher Hg-tolerance is observed along the positive direction of PC1 components that explains 45% (PC1-A) and 50% (PC1-B) of the total variance. As can be clearly observed in Figure 7A, control and treated leave samples of each variety are located close to each other in the multivariate space, suggesting that the variability between varieties is greater than the variability caused by the treatment. This behavior suggests that the treatment with Hg does not cause greater differences in the accumulation of leave flavonoids than those due to the differences between varieties in basal state. Leave samples distributed in the positive side of PC1 axis (L1 and L3) were associated with tolerance, whereas the sample with the most negative contribution on the PC1 axis (L4) is associated with the lowest tolerance.

In roots (Figure 7B), greater differences were observed in the accumulation of flavonoids in response to Hg stress. Tolerant cultivars under Hg-treatment (R1-Hg, R2-Hg, and R3-Hg) present a positive contribution in the PC1 axis, located in the tolerance zone where the Hg tolerance variable exhibit higher weight. On the contrary, both control and treated root samples of the less tolerant variety (R4-C and R4-Hg) showed strong negative contributions in PC1. Although R1, R2, and R3 are tolerant samples, their response to Hg-treatment was shown to be different in terms of flavonoids accumulation. This behavior might be explained due to their differences in the basal profile of flavonoids. Thus, R1-Hg is slightly displaced from R1-C sample, both located in positive PC1 component, indicating similar flavonoids accumulation in the basal state and under Hg stress conditions. However, R2-Hg and R3-Hg undergo a more pronounced displacement from their basal state (R2-C and R3-C in the negative PC1) toward positive contributions in the tolerance zone. This behavior evidences deeper changes in the flavonoids metabolic profile of R2 and R3 samples that allow them to tolerate Hg-stress, whereas R1 undergo slight modifications in response to Hg stress. The loading plot (Supplementary Figure 1) suggest that Hg-Tolerance might be associated to the accumulation of formononetin MalGlc (Form-G03), tricin GlcAGlcA (Tric-G03), and afrormosin Glc II (Afror-G02), among others, in leave samples, whereas the aglycones such as afrormosin, baidzein, biochanin A, and formononetin are associated with tolerance to Hg stress in root samples.

Another multivariate data analysis was also carried out based on the flavonoids glycosylation degree of the target control and Hg-treated varieties. The resulting PCA bi-plots are graphically displayed in Figure 8, where the first two principal components (PC1, PC2) explained the 78 and 69% of the total variance for leaves and roots, respectively. Sample grouping and distribution in the multivariate space showed similarities with the previous analysis based on the individual flavonoids profile. While control and treated leave samples of each variety grouped together, greater differences were observed between control and Hg-treated roots samples. Since the variability between varieties is greater for leaves than the variability caused by the treatment, no association could be done between flavonoids glycosylation and Hg-stress. However, a trend from lower to higher Hg-tolerance was observed along the positive direction of PC1 components that explains 57% (PC1-A) of the variability (Figure 8A). Tolerant L1 and L3 varieties were placed on the positive side of PC1, whereas the less tolerant L4 variety exhibited the most negative contribution in PC1. This trend is even more clear in Figure 8B, where Hg-treated roots (R1-Hg, R2-Hg, and R3-Hg) shifted with respect to the untreated controls toward the positive side of PC1, that is, the tolerance zone. Thus, under Hg stress, Hg-tolerant root samples showed a characteristic glycosylation pattern with positive contributions in the tolerance zone, which seems to be more associated with the accumulation of aglycones and low-glycosylated flavonoids (monoglycosylated) with higher contribution in positive PC1.

## Discussion

Several profiling studies, mainly based on HPLC-MS/MS methods have investigated the flavonoid components of *M. truncatula* (Jasiński et al., 2009; Marczak et al., 2010; Staszków et al., 2011). GC-MS was also employed after enzymatic hydrolysis of conjugate flavonoids to assign the sugar stereochemical configuration (Farag et al., 2007; Schliemann et al., 2008). Complementary spectroscopic techniques, including UV and nuclear magnetic resonance (NMR) detectors were also used to accurately elucidate their structures, increasing the number of identified flavonoid components (Kowalska et al., 2007; Jasiński et al., 2009). These advances allowed the investigation of the changes in the profile of flavonoid accumulation in *M. truncatula* under biotic stress (Jasiński et al., 2009), among others. The CID-MS/MS allowed us to define a pattern of flavonoid glycosides that was mainly characterized by the cleavages of consecutive glycosidic bonds or between sugar and the flavone moieties. We also identified product ions with charge retained on glucuronic acid moieties acylated with ferulic or coumaric acid. This typical fragmentation suggests O-glycosidic bonding (Jasiński et al., 2009), whereas the position of sugar acylation, as well as those of glycosidic bonds between the aglycone and sugar or sugars have been reported in LC/MS-based structural elucidation works, where NMR spectra have been included (Kowalska et al., 2007; Jasiński et al., 2009). In leaves, our results are in line with previous works, reporting contents of flavone glycoconjugates in relative concentrations greatly exceeding those of isoflavones in *M. truncatula* leaves (Jasiński et al., 2009). The prevalence of glycoconjugates substituted with glucuronic acid moieties that we observed related to aglycones is supported by earlier studies performed on leaves from *M. truncatula* (Staszków et al., 2011). The presence of a higher concentration of isoflavone derivatives in root tissues is most probably due to the fact that intense interactions with other organisms, mainly rhizobia, but also other bacteria, fungi, insects, and other plants occur in the rhizosphere. Many plant species use isoflavonoids as signaling and defense compounds in their interactions with both symbionts and pathogenic microbes. In addition, roots of leguminous plants exude specific flavonoids into the rhizosphere, which act as chemical attractants for symbiotic nitrogen-fixing bacteria (Oldroyd et al., 2005; Jasiński et al., 2009).

The role of flavonoids in the response to heavy metals, specifically mercury, has not been investigated to date. To the best of our knowledge, the present study is the first metabolomic analysis of *M. truncatula* focused on flavonoids accumulation in response to mercury stress.

Under environmental stresses, plant cells initiate gene expression programs at the transcriptional level, which regulate metabolite accumulation to adapt to the new conditions (Hu et al., 2022). In a recent GWAS on mercury tolerance in *M. truncatula* (Paape et al., 2022), we identified a UGT gene as a candidate gene within the proximity to the top SNP related to Hg tolerance. There were other UGT genes in the same region of chromosome 2, whose substrates are unknown. These results suggest that flavonoids and their glycoconjugates can have an important role in the response to Hg. In the present work, metabolomic data can help to uncover the molecular mechanisms at the metabolite expression level, underlying flavonoid accumulation in *M. truncatula* in response to Hg stress.

Thus, metabolomic data analyses suggest that significant variations in the flavonoids profile of the studied varieties are altered upon Hg treatment, affecting differently to leaf and root tissues as described above. In tomato plants subjected to heavy metal stress (zinc and copper), an increase in flavonoid accumulation could be observed; however, in this case the highest accumulation occurred in leaves compared to roots (Badiaa et al., 2020). An increase of flavonoid content in cotyledons occurs in buckwheat seedlings subjected to heavy metal stress. Interestingly, heavy metal-tolerant buckwheat cultivars present higher flavonoid accumulation levels than sensitive cultivars (Horbowicz et al., 2013).

**FIGURE 9:**
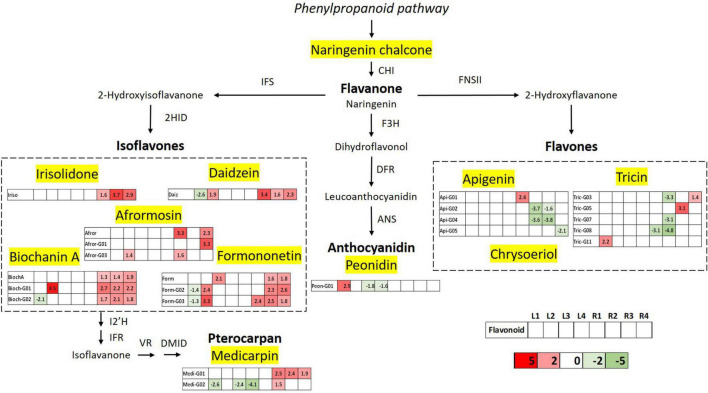
Schematic diagram of the flavonoid biosynthesis pathway integrating the metabolomics results of differentially accumulated flavonoids upon Hg stress in *Medicago truncatula* leaf and root samples. Differentially expressed flavonoids are displayed in a color code based on the Log_2_FC value; downregulated flavonoids (Log_2_FC < 1.33) are indicated in green and upregulated components (Log_2_FC > 1.33) are shown in red. CHI, chalcone isomerase; IFS, isoflavone synthase; F3H, flavanone 3 β-hydroxylase; 2HID, 2-hydroxyisoflavanone dehydratase; DFR, dihydroflavonol-4-reducatse; FNSII, flavone synthase; ANS, anthocyanidin synthase; I2’H, isoflavone 2’-hydroxylase; IFR, isoflavone reductase; VR, vestitone reductase; and DMID, dihydroxy-40-methoxy-isoflavanol dehydratase.

The flavonoid biosynthetic pathway has been clearly delineated in some model plants like *M. truncatula.* On the basis of this model, a pathway diagram was constructed showing the differentially expressed flavonoids in the studied varieties of *M. truncatula* leaf and root samples. A total of 11 classes of identified flavonoids were mapped, along with their related genes and metabolic precursors, in the biosynthetic pathway (Figure 9). Differentially expressed aglycones and glycosylated derivatives are displayed in boxes, one for each target sample (L1, L2, L3, L4, R1, R2, R3, R4), using a color code from green (downregulated) to red (upregulated) based on Log_2_FC value. The very first step in the phenylpropanoid pathway is catalysed by the enzyme phenylalanine ammonia lyase (PAL), and *PAL* expression has been reported to be induced by heavy metals; however, it is possible that this induction leads to enhanced phenolic acids accumulation, without necessarily affecting flavonoid biosynthesis (Ma et al., 2016; Berni et al., 2019). In the diagram presented in Figure 9, we consider, naringenin chlacone the first compound in the flavonoid biosynthetic pathway. Naringenin chalcone is transformed into naringenin – a core metabolite of the flavonoid pathway- *via* the enzymatic activity of chalcone isomerase. The metabolomic data did not show deregulation at this point of the route, which suggests that the phenylpropanoid pathways is not affected. The resulting flavanone naringenin, serves as a substrate for isoflavone synthase (IFS), flavone synthase (FNS), and flavanone 3-hydroxylase (F3H) to produce isoflavones, flavones and (dihydro)flavonols, respectively, (Gholami et al., 2014). Under heavy metal stress, flavonoid accumulation in plants is typically induced *via* the transcriptional regulation of biosynthesis genes (Naing and Kim, 2021 and references therein).

The first step of isoflavonoid biosynthesis begins with an oxidative aryl migration from C-2 to C-3, catalyzed by IFS enzyme to form the 2-hydroxyisoflavanone intermediates, and subsequent dehydration by 2HID to generate isoflavones like genistein and daidzein. This “undecorated” isoflavones are precursors of the 4′-O-methylated forms biochanin A and formononetin, respectively, which are major isoflavonoid aglycones detected in *M. truncatula* roots (Figure 3). Further modifications in the A-ring, generate aglycones such as afrormosin and irisolidone, the first one derived from formononetin. The metabolomic results reveal that isoflavone biosynthesis is the most affected metabolic route in flavonoids pathways upon mercury stress. The five detected isoflavone aglycones show upregulated levels in at least three of the studied varieties, mainly in root samples, suggesting increased levels in the activity of IFS and 2HID enzymes. Glycosylated forms of biochanin A and formononetin were also found to be significantly upregulated, evidencing the metabolic interconnection and relevance of these isoflavones in the secondary metabolism of *M. truncatula.*

Pterocarpans form a class of isoflavonoid derivatives in which the B-ring is coupled to the C-ring *via* a furan ring. The biosynthesis of medicarpin, the pterocarpan detected in the *M. truncatula* root samples, starts with the conversion of formononetin to 20- hydroxyformononetin, catalyzed by the P450 enzyme I2′H. The upregulated levels of the glycosylated derivatives of medicarpin is consistent with the altered levels of its isoflavone precursor formononetin and glucoside and malonylglucoside forms. Peonidin glucosides are the only anthocyanidin-type flavonoids identified in the studied *M. truncatula* samples, and accumulated mainly in leaves. The biosynthesis of anthocyanidins and their precursor flavonols is mainly controlled by B-ring hydroxylation enzymes such as F3H, followed by dihydroflavonol-4-reductase and anthocyanidin synthase that converts the leucoanthocyanidins to the corresponding anthocyanidins. This branch of the pathway shows deregulation in the glycosylation levels of peonidin, suggesting and impairment in the anthocyanidin glycosylation process of leave tissues.

The biosynthesis of flavones is the second most affected route in *M. truncatula*, as a result of the Hg treatment, occurring mostly in leaf tissues where they are mainly accumulated. Flavones biosynthesis from flavanones occurs by introducing a double bond between C-2 and C-3, that is catalyzed by FNS *via* 2-hydroxyflavanone intermediate. Three types of flavones, namely apigenin, tricin, and chysoeriol have been detected in *M. truncatula* samples in their glycoconjugated forms. Despite being major flavonoids in leaves, apigenin, and tricin glycoside levels were shown to be unaltered in the aerial part, upon Hg stress. On the contrary, the levels of these conjugated flavonoids were mainly deregulated in root tissues.

Flavonoids can undergo different modifications, including acylations, hydroxylations, methoxylations, prenylations, or glycosylations (Dixon and Pasinetti, 2010). Glycosides are the most common form of flavonoid derivatives. From the metabolomic results, it is clear that almost all detected flavonoids in the analyzed *M. truncatula* samples are accumulated in their conjugated forms with one or more sugar moieties, and very often as acylated glycoconjugates. Glycosylation enhances the water solubility of flavonoids and is critical for the transport and sequestration of these compounds in the vacuole (Behr et al., 2020 and references therein). Glycosylation appears to be the prerequisite for flavonoids to be transported into the vacuole, whereas aglycones can be transported out of the cell (Zhao, 2015). The high upregulated levels of conjugated isoflavones detected in roots samples, mainly monoglycosylated, suggest an activation of the storage mechanism of these flavonoids in response to Hg treatment. In addition, although in minor extent, the upregulation of the isoflavonoids aglycone suggest some degree of mobilization of free isoflavones to be transferred to the cytosol and possibly secreted out of the cell, as flavonoids can also be secreted to immobilize heavy metals in the rhizosphere (Cesco et al., 2010). This delicate equilibrium between mobilization of free aglycones by conjugates hydrolysis and sequestration by glycosylation can play a crucial role in the transport and sequestration of heavy metals like mercury, considering that flavonoids can act as metal chelators (Gholami et al., 2014). The transport and trafficking of flavonoids has been reported to be finely regulated (Zhao and Dixon, 2010; Zhao, 2015).

In conclusion, in the present work, a comprehensive metabolomic data analysis was carried out to uncover the molecular mechanisms at the metabolite expression level, underlying flavonoid accumulation in *M. truncatula* in response to Hg stress. The metabolomic data analysis suggest that significant variations in the flavonoids profile of the studied varieties are induced upon Hg treatment, affecting differently to leaf and root tissues. The results showed a total of 46 identified flavonoids compounds, classified into five different flavonoid families: anthocyanidins, flavones, isoflavones, pterocarpan flavonoids, and flavanone, along with their respective glycoconjugate derivatives. Flavones and isoflavones accounted for 81–93% and 77–88% the total flavonoids in leaves and roots, respectively; indicating that they are the major flavonoid classes in *M. truncatula*, and evidencing the differential composition between the aerial part and root tissues. The case of LR1, the most tolerant variety, is noteworthy, as this cultivar presents practically the same flavonoid accumulation and profile in control and stress conditions. This could indicate that its basal flavonoid profile is capable to neutralize the effects of Hg stress or else, alternative mechanisms are sufficient to cope with Hg stress and no additional contribution of the flavonoid antioxidant and chelating activity is required.

In general, under Hg stress, specific groups of flavonoids were differentially expressed in roots and leaves. Thus, malonylglucosides of biochanin A, formononetin and medicarpin followed by the isoflavone aglycones biochanin, daidzein, and irisolidone were strongly deregulated in *M. truncatula*. These results are in line with the outcomes form the multivariate PCA analysis, suggesting that Hg-tolerance might be associated to the accumulation of aglycones such as formononetin, daidzein, biochanin A, and their malonylglycosylated derivatives. Our work reveals a clear mobilization of flavonoid compounds (increase in flavonoid production) in response to stress generated by Hg, mainly in roots.

## Data Availability Statement

The original contributions presented in this study are included in the article/Supplementary Material, further inquiries can be directed to the corresponding authors.

## Author Contributions

JP and ML conceived of the study. AC, EI, ML, and JP supervised the experimental work. GA-R and AS carried out the experiments. GA-R wrote the first draft of the manuscript. AS and JP wrote sections of the manuscript. TP aided in interpreting the results and provided critical feedback. All authors contributed to manuscript revision, read, and approved the submitted version.

## Conflict of Interest

The authors declare that the research was conducted in the absence of any commercial or financial relationships that could be construed as a potential conflict of interest.

## Publisher’s Note

All claims expressed in this article are solely those of the authors and do not necessarily represent those of their affiliated organizations, or those of the publisher, the editors and the reviewers. Any product that may be evaluated in this article, or claim that may be made by its manufacturer, is not guaranteed or endorsed by the publisher.

## Footnotes

1
www.metaboanalyst.ca
