# Supplementary material for: Flavonoid Accumulation Varies in Medicago truncatula in Response to Mercury Stress
Source: Front Plant Sci. 2022 Jul 7;13:933209. doi: 10.3389/fpls.2022.933209 (PMC9301243; doi:10.3389/fpls.2022.933209)
Supplement: Supplementary file 1 [file Data_Sheet_1.PDF]

Table S1. Relative abundance (%) of flavonoid families in *Medicago truncatula* leaves (L) and roots (R).

**Flavonoid family**	**L1**	**L2**	**L3**	**L4**	**R1**	**R2**	**R3**	**R4**
Anthocyanidin_Peonidin	1.7	0.3	0.9	0.4	0.0	0.0	0.1	0.0
Flavanone	1.9	3.1	2.2	1.2	0.0	0.0	0.0	0.0
Flavone_Chrysoeriol	5.5	4.6	1.7	5.1	0.0	0.0	0.0	0.0
Flavone_Apigenin	21.5	43.3	8.7	65.4	0.0	0.2	0.2	1.0
Flavone_Tricin	65.6	48.0	81.3	26.9	0.4	1.0	0.5	1.0
Isoflavone_Formononetin	1.6	0.2	0.8	0.3	76.7	82.3	78.6	87.6
Isoflavone_Biochanin	0.1	0.0	0.2	0.1	1.7	2.0	1.6	0.9
Isoflavone_Daidzein	0.0	0.0	0.1	0.0	1.1	0.4	0.2	0.6
Isoflavone_Afrormosin	2.0	0.5	4.0	0.5	10.3	8.7	11.8	6.0
Isoflavone_Irisolidone	0.1	0.0	0.1	0.0	5.1	3.8	1.3	0.7
Pterocarpan-Medicarpin	0.1	0.0	0.0	0.1	4.6	1.7	5.7	2.0

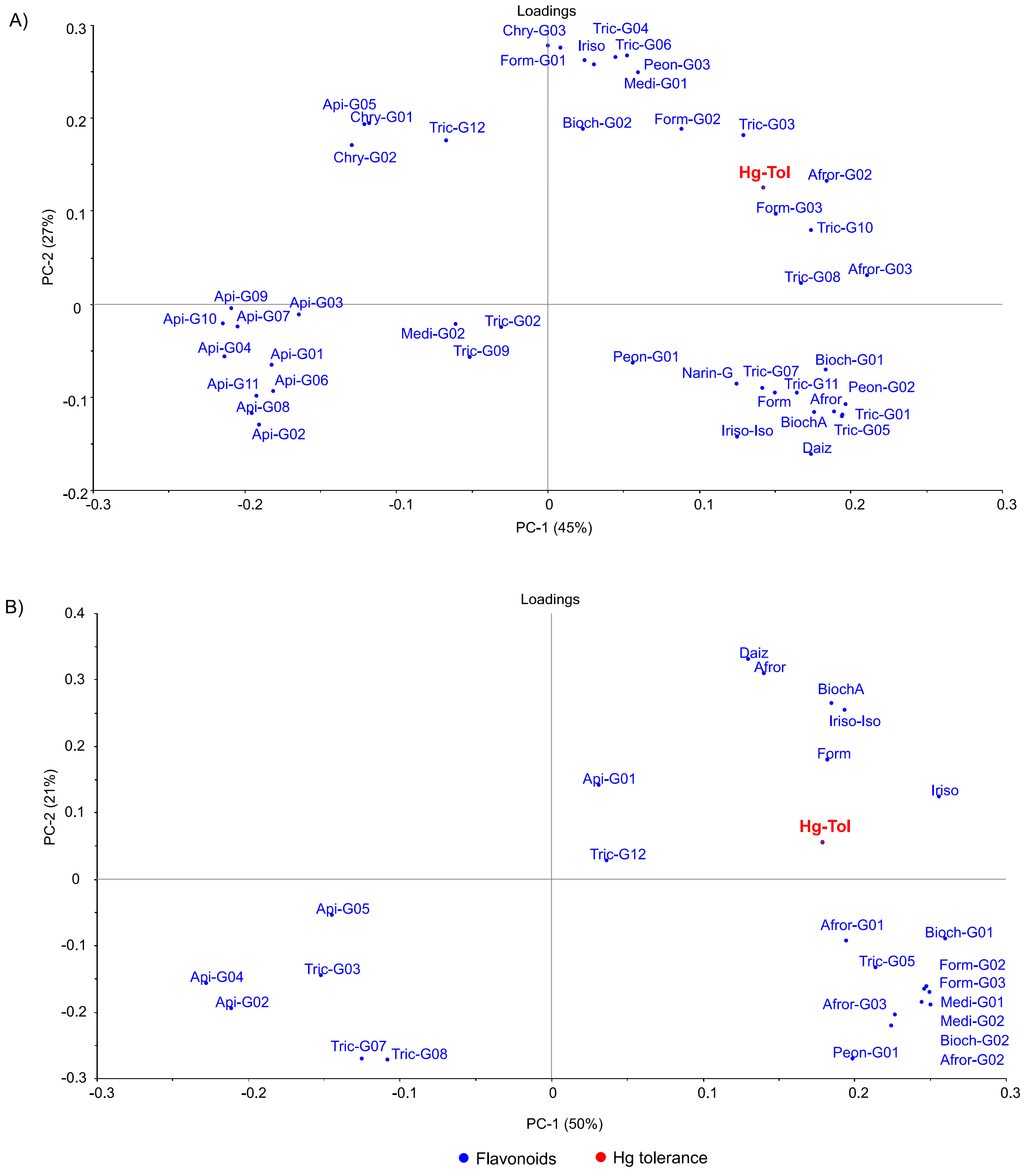

Figure. S1. PCA loading plots showing the distribution of the studied variables: flavonoids composition (blue) and Hg tolerance (red), in control and Hg-treated varieties of *Medicago truncatula*. A) Leaves and B) Roots.
